# Finding the Correct Partner: The Meiotic Courtship

**DOI:** 10.6064/2012/509073

**Published:** 2012-08-08

**Authors:** Tomás Naranjo

**Affiliations:** Departamento de Genética, Facultad de Biología, Universidad Complutense de Madrid, 28040 Madrid, Spain

## Abstract

Homologous chromosomes are usually separated at the entrance of meiosis; how they become paired is one of the outstanding mysteries of the meiotic process. Reduction of spacing between homologues makes possible the occurrence of chromosomal interactions leading to homology detection and the formation of bivalents. In many organisms, telomere-led chromosome movements are generated that bring homologues together. Additional movements produced by chromatin conformational changes at early meiosis may also facilitate homologous contacts. Organisms used in the study of meiosis show a surprising variety of strategies for homology detection. In dipterans, homologous chromosomes remain paired throughout most of development. Pairing seems to arise as a balance between promoter and suppressor pairing genes. Some fungi, plants and animals, use mechanisms based on recombinational interactions. Other mechanisms leading to homology search are recombination-independent and require specialized pairing sites. In the worm *Caenorhabditis elegans*, each chromosome carries a pairing center consisting of a chromosome-specific DNA-protein complex, and in the fission yeast *Schizosaccharomyces pombe*, the *sme2* locus encodes a meiosis-specific non-coding RNA that mediates on homologous recognition. In addition, mismatch correction plays a relevant role, especially in polyploids, which evolved genetic systems that suppress pairing between non-homologous related (homoeologus) chromosomes.

## 1. Introduction

Meiosis is a central process in the life cycle of all sexually reproducing organisms that evolved to compensate for the duplication of the chromosome number produced at fertilization and to generate new combinations between parental alleles that boost genetic diversity. Germ cells enter meiosis having replicated their chromosomes and, by executing two successive cellular divisions with no intervening DNA replication, produce haploid gametes. Germ cells of diploid organisms initiate meiosis containing two chromosome sets, one inherited from each parent. Homologous chromosomes are partitioned in the first division (reductional division) and sister chromatids segregate away from each other in the second division (equational division). For proper chromosome segregation, homologues interact and generate stable associations during prophase I that maintain them linked in a bivalent configuration until metaphase I. The cytological structures that link each homologous pair at metaphase I are called chiasmata. Cohesion of sister chromatids cooperates with chiasmata in providing stability to the bonds between each homologous pair through metaphase I. Breakdown of the nuclear envelope allow that microtubules interact with sister kinetocores, which become oriented to the same pole of the spindle. Dissolution of sister chromatid cohesion, except in the pericentromeric region, enables chiasma resolution and chromosome segregation at anaphase I. 

**Figure 1:**
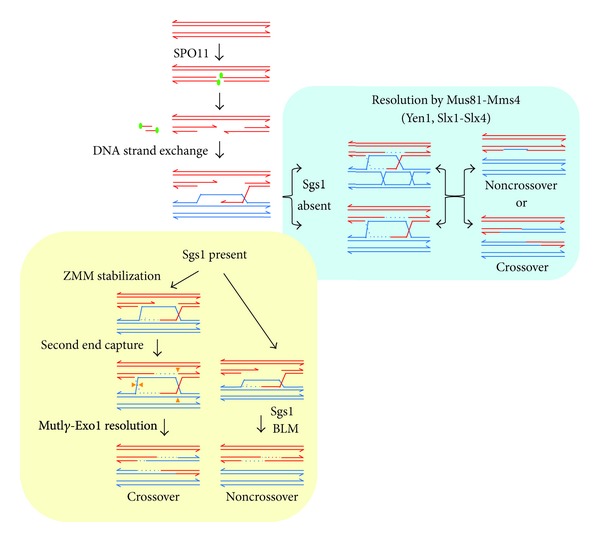
Homologous recombination during meiosis. Meiotic recombination begins with a break made by Spo11 in one of the two double-strand DNA molecules of one chromosome. The 5′ ends of this break are resected leaving tails of 3′ single-strand DNA. When the 3′ ssDNA invade a homologous chromatid, the presence of the Sgs1 protein can reverse the strand inversion intermediates to form a noncrossover outcome or promotes association of some legitimate strand invasion intermediates with the ZMM protein complex. These molecules are resolved in a crossover, mainly by MutL*γ*-Exo1. Molecules formed in the absence of Sgs1 are the target of Mus81-Mms4, Yen1, and Slx1–Slx4, which resolve in a random mode to give both crossover and noncrossover outcomes.

Chiasmata are formed after culmination of three major processes initiated in early prophase I, homologous pairing (i.e., an interaction of chromosomes that results in the close apposition of homologues along their entire length), synapsis (i.e., the formation of a proteinaceous synaptonemal complex structure between each homologous pair), and crossing over (i.e., a reciprocal exchange of genetic material between homologous chromatids). A crossover and a noncrossover (nonreciprocal exchange) represent the two possible outcomes that the homologous recombination machinery follows to repair one DNA double-strand break (DSB) generated at the initiation of meiosis. Crossovers involve the reciprocal exchange of sequences flanking the repair sites and noncrossovers (also referred to as gene conversions), the transfer of local information, spanning a few hundred base pairs, from one homologue to the other. The majority of DSBs are destined to become noncrossover products. The main steps of the recombination mechanism have been uncovered in yeasts and are conserved in other eukaryotes [1]. Meiotic recombination is initiated by a conserved topoisomerase-like enzyme, Spo11, which introduces programmed DSBs into the genome. Spo11 cuts DNA via a topoisomerase-like reaction to generate covalent protein-DNA linkages to the 5′ DNA ends on either side of the break. The enzymatic machinery that repairs DSBs is orchestrated in such a way that the DNA sequences preferred as template are those of the homologous chromosome while participation of the sister chromatid is inhibited. Following Spo11 removal, DSB ends then undergo nucleolytic resection of the 5′ strands to produce 3′ single-stranded tails. One of these tails can then invade an intact double-strand DNA of the homologous chromosome. In most eukaryotes, this reaction, which is known as strand invasion, requires the action of two recombinases, Rad51 and Dmc1, which act in association with other proteins [2]. The initial strand invasion intermediates can be further processed in different ways, with different recombination product outcomes (Figure 1). If the single DSB end that invaded the homologous partner, after priming DNA synthesis, is displaced and anneals with the other DSB end, a noncrossover is produced. This process is called synthesis-dependent strand annealing (SDSA). An alternative pathway leads to stabilization of strand invasion intermediates and capture of the second DSB end that primes DNA synthesis. Ligation generates a double Holliday junction joint molecule intermediate, which is resolved yielding a crossover product. Crossover and noncrossover recombinational interactions diverge at the leptotene-zygotene transition, prior to the formation of extensive strand-exchange intermediates [3, 4]. The Sgs1 protein of the budding yeast *Saccharomyces cerevisiae*, a homologue of the mammalian BLM helicase that maintains genome stability by preventing accumulation of aberrant recombination intermediates, is a central regulator of recombination pathway choice in normal meiosis [5, 6]. Sgs1 controls meiotic recombination by preventing accumulation of unregulated joint molecules intermediates. Sgs1 displaces the invading strand of joint molecules to form noncrossovers and prevents the accumulation of multichromatid joint molecules. In addition Sgs1 channels some other joint molecules to interact with a group of meiosis-specific proteins referred to as the ZMM family, which are required to form stable joint molecules and double Holyday junction intermediates. These ZMM-protected recombination intermediates are later, at the end of pachytene, resolved as crossovers by a multiprotein factor containing the nuclease Exo1 and the MutL*γ* complex Mlh1–Mlh3, which is activated by the kinase polo-like Cdc5. Joint molecules derived of single-strand invasion that escape the control of Sgs1, or those produced in its absence, form often multichromatid intermediates, which yield both crossover and noncrossover products, principally under the action of the Mus81-Mms4 (MUS81-EME1 in human) resolvase complex that also requires Cdc5 and in a minor degree by the action of Yen1 (GEN1) or Slx1–Slx4 (BTBD12/SLX4). Other proteins involved in the crossover and noncrossover pathways are reviewed by [7].

**Figure 2:**
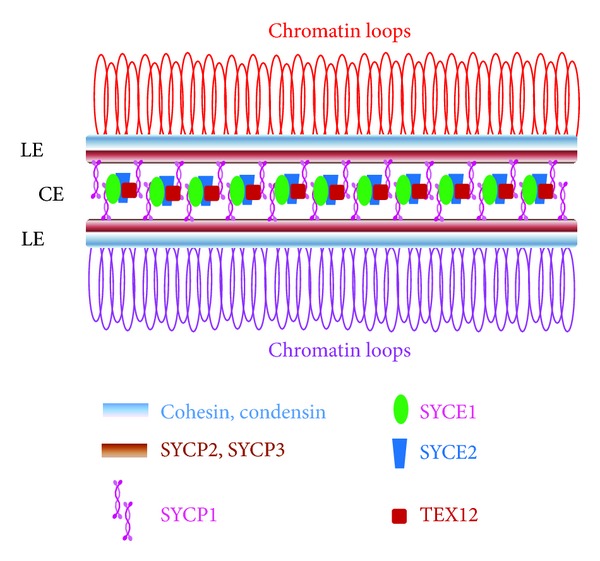
Cartoon showing the structure and major components of the lateral elements (LEs) and central element (CE) of the synaptonemal complex in mammals. Chromatin loops of each chromosome of the homologous pair emanate from each LE.

 The synaptonemal complex is a proteinaceous matrix that normally forms between each homologous pair, reinforcing their interaction, and occurs in most organisms with sexual reproduction. The synaptonemal complex assembly starts during premeiotic S phase and early meiotic prophase with the formation of a single proteinaceous axis, the axial element, along the two sister chromatids of each chromosome. In early prophase, the axial element connected to a pair of sister chromatids closely associates at *≈*100 nm along its length with the axial element of the homologous chromosome; they are then named lateral elements. The longitudinal apposition of homologues is mediated by the installation of numerous transverse filaments and the formation of the central element. The synaptonemal complex is the ladder-like structure formed by the two lateral elements, the transverse filaments and the central element (Figure 2).

Components of the synaptonemal complex have been identified in different species (reviewed by [8, 9]). The cohesin protein complex is required to establish cohesion between sister chromatids during DNA replication in somatic cells. Cohesins are component of the axial/lateral element but some members of the mitotic complex are replaced by meiosis-specific paralogues. Condensin proteins also form part of the axial/lateral elements and are necessary for axial length compaction and chromosome individualization, similar to their effects on mitotic chromosome condensation. Other proteins, such as SYCP2 and SYCP3 in mouse, Hop1 and Red1 in budding yeast, HIM-3 in *C. elegans,* Asy1 in *Arabidopsis thaliana*, and ORD in *Drosophila*, are members of the lateral element. In addition to their role on chromatin condensation, axial/lateral elements are involved in the alignment of homologues. The cohesion/condensing chromosomal axis and/or associated proteins, such as Hop1p and HIM-3, are involved in the assembly of the transverse filaments. Regulation of the mode in which DSBs are repaired to crossover or noncrossover products is also mediated by some lateral elements proteins. Proteins that form the transverse filaments have been identified in several species. These include Zip1p in *S*. *cerevisiae*, SYCP1 in mammalian, C(3)G in *Drosophila*, SYP-1 and SYP-2 in *C*. *elegans,* and ZYP1 in *Arabidopsis*. These proteins have terminal C and N globular domains separated by an extended coiled-coil region. Parallel protein dimmers associate through the interdigitating N-termini and form a tetramere that spans the gap between lateral elements. In mouse, stable transverse filaments require association of SYCP1 tetrameres and SYCE1. Proteins SYCE1 and SYCE2 locate at the central element. Transverse filaments and central element protein are required for completion of the recombinational interaction crossover pathway.

 Interaction between homologous chromosomes mentioned previously requires their positioning in close physical proximity into the nucleus. The term alignment usually refers to the topological disposition of chromosomes with the arms arrayed in parallel. Pairing refers to close and stable chromosome interactions that involve multiple points and result in intimate association of homologues along their entire length. Although early studies showed that homologous chromosomes are not paired at leptotene [10] an ordered premeiotic interphase nucleus with presynaptic homologous chromosome alignments as part of that order was proposed [11, 12]. However, studies carried out in different organisms contradict this proposal. In fungi with zygotic meiosis, such as *Sordaria, Neurospora,* and *Coprinus*, homologous chromosomes are in different nuclei before fertilization. After karyogamy, homologues remain separated until the commencement of interactions [13]. Among plants, chromosome painting was used to identify the position of two maize homologues added to oats [14] as well as two homologues of rye added to wheat [15]. Such painted chromosome pairs are occupying separated territories in most nuclei at premeiotic interphase, ruling out a role of premiotic arrangement in the meiotic partner selection. The spatial organization of the mammalian genome in somatic cells has attracted growing attention the last decade. The mammalian genome is organized in a structured nucleus in which the folding and relative positioning of the chromosomes constitutes a high-order regulatory mechanism of gene expression [16]. Mammalian chromosomes are organized in discrete, nonoverlapping chromosome territories, with those of homologues usually not adjacent [17]. The arrangement of human chromosome pair 1 and mouse chromosome pair 8 in spermatogonia denotes absence of premeiotic pairing [18]. Only in organisms like Dipterans, homologous chromosomes are paired throughout the life cycle (somatic pairing). The behavior of tagged chromosomes with the GFP-lac repressor protein during male *D. melanogaster* meiosis indicated that the pairing events observed in the first meiotic division were a continuation of premeiotic pairings and not the result of meiosis-specific mechanisms [19]. Thus, with the exception of Dipterans, homologous pairing is promoted by mechanisms that reduce gradually the physical separation between homologues and discriminate homologous and nonhomologous interactions until culminate in a close and stable juxtaposition of homologues.

The mode by which homologous chromosomes come into close spatial proximity, recognize each other, and undergo stable interactions is one of the mechanisms most poorly understood of the meiotic process. Unraveling the genetic and molecular basis concerning the homology search and detection represents the aim of many current research works in different organisms. In this paper, I will concentrate on cellular activities that bring homologues together and aid to detect homology distinguishing marks, which culminate in the formation of homologous bivalents.

## 2. Telomere-Led Chromosome Movements Coming Near Homologous Pairs

A feature of the nuclear architecture at premeiotic interphase that has been related to meiotic pairing is the distribution and orientation of centromeres and telomeres. In mitotically active cells of many species, chromosomes retain during interphase the geography of the previous anaphase, that is, the Rabl configuration, with centromeres concentrated at one side of the nucleus and telomeres fanned out on the opposite side. This chromosome polarization extends to premeiotic cells, especially in species with large genomes [20], and ensures that homologous segments, although situated in separated territories, maintain similar distances to the nuclear poles, which would minimize the chromosome movements during homologous pairing [21].

In many organisms, telomeres of all chromosomes initiate a nonrandom movement at the entrance of meiosis that causes their progressive grouping to culminate in a tight cluster. This suprachromosomal configuration, the so-called bouquet, is consolidated concomitant with the initiation of alignment, pairing and synapsis, particularly at regions close to the chromosome ends, hence, it is currently considered as a meiotic-specific structure involved in homologous chromosome interactions [22–27]. Other roles, including bivalent interlocking resolution and regulation of recombination, have also been suggested for the bouquet structure [28]. The formation of the bouquet arrangement is the result of three interdependent events: attachment of telomeres to the nuclear envelope, clustering of chromosome ends, and cytoskeleton-mediated telomere movement (reviewed by [29]).

Molecular interactions responsible of the attachment of chromosome ends to the nuclear envelope require specifically the presence of functional telomere repeats. Telomere shortening in telomerase-deficient mice impairs synapsis and decreases the recombination frequency [30]. The de novo presence of telomere repeats in the centromeric end of telocentric chromosomes generated by centromere misdivision of two-armed chromosomes changes the dynamics of the centromere. These telomere repeats exert a cis-dominant effect on the centromere, which can incorporate to the telomere cluster while original centromeres remain at the opposite pole of the nucleus [15]. When the telomeric sequences are intercalarily located, as it happens in a maize ring chromosome, they also associate with other telomeres and make the chromosome to enter the bouquet [31].

Attachment of telomeres to the inner surface of the nuclear envelope requires the presence of constitutive chromosome ends associated proteins, such as Taz1, Rap1, and Rik1, and meiotic specific proteins that form a bridge between telomeres and the nuclear envelope. These proteins include Ndj1, Mps3, and Csm4, in budding yeast [32, 33] and Sad1, Kms1, Bqt1 and Bqt2 in fission yeast [24, 34]. Some of these meiotic proteins, such as Sad1 and Kms1, are transmembrane proteins with a SUN (Sad1-Unc-84) domain and a KASH (Klarsicht/ANC-1/Syne homology) domain, respectively [35]. The Sad1 protein is oriented to the nucleoplasm and linked to the telomeres by the Bqt1-Bqt2 complex. The SUN domain of Sad1, located at its C-terminus, is thought to interact physically through the intermembrane space with the KASH domain of the Kms1 protein integrated in the outer membrane. The KASH domain corresponds to a short region of the C-terminus of the protein and the large N-terminus tail extends into the cytoplasm and connects the outer membrane with dynein, a cytoplasmic minus-end-directed microtubule motor. Mutations in Sad1 or Kms1 severely reduce crossover and impair homolog segregation, while mutations in dynein cause more subtle defects. SUN and KASH domain proteins play a conserved role by establishing transient linkages between chromosome ends and cytoskeletal components across the intact nuclear envelope. The Mps3 protein of budding yeast is other member of the SUN domain protein family that is linked to the telomeres by Ndj1. However, no KASH domain protein capable of liking Mps3 to some cytoskeletal component has been identified. In *C. elegans*, the SUN-1 nuclear inner membrane protein interacts with the KASH domain protein ZYG-12 of the outer layer to link chromosomes, via their pairing centers, to microtubules network and cytoplasmic dynein [36]. In mutants of genes *sun-1* or *zyg-12*, homologous chromosomes do not pair properly and synapse promiscuously. The inner nuclear membrane protein SUN1 specifically associates with telomeres between the leptotene and diplotene stages during meiotic prophase I in mice [37]. Disruption of *sun1* prevents telomere attachment to the nuclear envelope, efficient homolog pairing, and synapsis. However, no KASH counterpart for SUN1 has been still reported. In *Arabidopsis,* double mutants for genes *sun1* and *sun2* show synapsis failure and decreased chiasma frequency (JL Santos, personal communication).

 Telomeres attached to the nuclear envelope associate first in a number of small aggregates, which undergo active movements to become associated in a tight cluster. Telomere association and telomere migration are two different steps in the bouquet consolidation as deduced from the results produced by the treatment of wheat premeiotic and meiotic cells with colchicine. This microtubule depolymerizing agent inhibits telomere migration and impairs synapsis but does not affect telomere association [38]. This association is most likely produced between telomeres attached to very near nuclear envelope sites, as in the case of the ends of the homologous arms of an isochromosome. These arms lie close to one another after the last premeiotic mitosis and display a normal level of pairing and chiasma formation even in the presence of colchicine [39].

 At the entrance of meiosis homologous chromosomes occupy spatially separated territories. Spacing between homologues may reach several *μ*m in organisms with a large genome; for example, the wheat nucleus at leptotene is *≈*24 *μ*m in diameter. Reduction of such a distance requires an active chromosome movement, which in addition should be properly oriented to bring homologous chromosomes into close proximity. Spacing between homologues falls into a much more reduced scale in the fission yeast with a small genome (13,8 Mb) and three chromosome pairs included in a cell that measures 3,5 *μ*m in diameter and 10 *μ*m in length. In this organism, meiosis occurs in the zygote after fusion of the two gametal nuclei, and homologues need to be replaced to become aligned. Alignment is reached by characteristic movements of the elongated nucleus, called a “horsetail” nucleus. The horsetail nucleus moves back and forth across the zygote for about 2 h. This period, corresponding to meiotic prophase, provides the only opportunity for chromosomes to pair and recombine with their homologous partners. Nuclear movements are mediated by astral microtubules, which radiate from the spindle-pole body (a microtubule-organizing center in fungi), and a dynein protein motor [40, 41]. During this process, the Sad1-Kms1 protein complex interacts with telomeres on the nucleoplasmic side and with the dynein protein motor on the cytoplasmic side. In this way, telomeres are moved by the driving force generated by the dynein motor on microtubules. Telomeres form a cluster close to the spindle-pole body, and this arrangement together with the oscillatory nuclear movement situates the homologues aligned.

In budding yeast, chromosome sorting and pairing produced in early meiosis is accompanied by rapid telomere-led chromosome movements in which telomeres cluster near the spindle-pole body to form the bouquet. These movements are mediated by telomere-attached proteins Ndj1 and Mps3 and by protein Csm4, which is not involved in the nuclear envelope-telomere attachment but in the transmission of the force generated by the cytoskeleton [33]. Telomere-led chromosome movements in budding yeast are not dependent of the dynein-microtubules complexes [42]; they are governed by actin filaments, since the inhibition of actin polymerization by latruculin B disrupt telomere clustering [43]. Bouquet formation is affected by colchicine in plant meiocytes. However, telomere movement is not mediated by cytoplasmic microtubules. A membrane-associated tubulin or tubulin-related proteins were suggested as the target of colchicine [44].

 It is generally thought that the bouquet formation facilitates homologous pairing by reducing the nuclear space where chromosomes have to move to find the homologous partner [27]. The bouquet formation implies that all telomeres, regardless of their position in the nuclear periphery, migrate to a small region of the nuclear envelope. This movement is most likely polarized by the position of the spindle-pole body in fission yeast. However, in organisms such as plants, without a defined microtubule organizing center, it is difficult to envisage how the movement of telomeres is oriented to converge in a region opposite the centromere pole. One can argue that the Rabl disposition facilitate the polarized movement. However, the convergent cytoskeleton forces operate throughout the complete nuclear surface since they are capable of clustering telomeres of telocentrics located in the centromere pole and those of two-armed chromosomes located in the opposite hemisphere [15]. Telomeres mobility can be affected by noncytoplasmic factors such as chromosomal conformation. This effect became apparent when the migration of the individual chromosome ends of a biarmed chromosome was followed in two different conformations, submetacentric chromosome, that is the chromosome arms have a very different length, and metacentric chromosome, that is, arms with a similar length. The unequal arms situation corresponds to the standard chromosome 5R of rye and the metacentric conformation arose by a large deletion produced in the long arm of this chromosome. The short arm is the same in both chromosome conformations, but its telomere reaches the telomere pole more often in the metacentric constitution than in the submetacentric constitution [45]. This variable behavior suggests that the presence of the long arm exerts some resistance to the movement of the short arm telomere or interferes in its attachment to the nuclear membrane.

## 3. Chromosome Movements Independent of Telomere Migration

**Figure 3:**
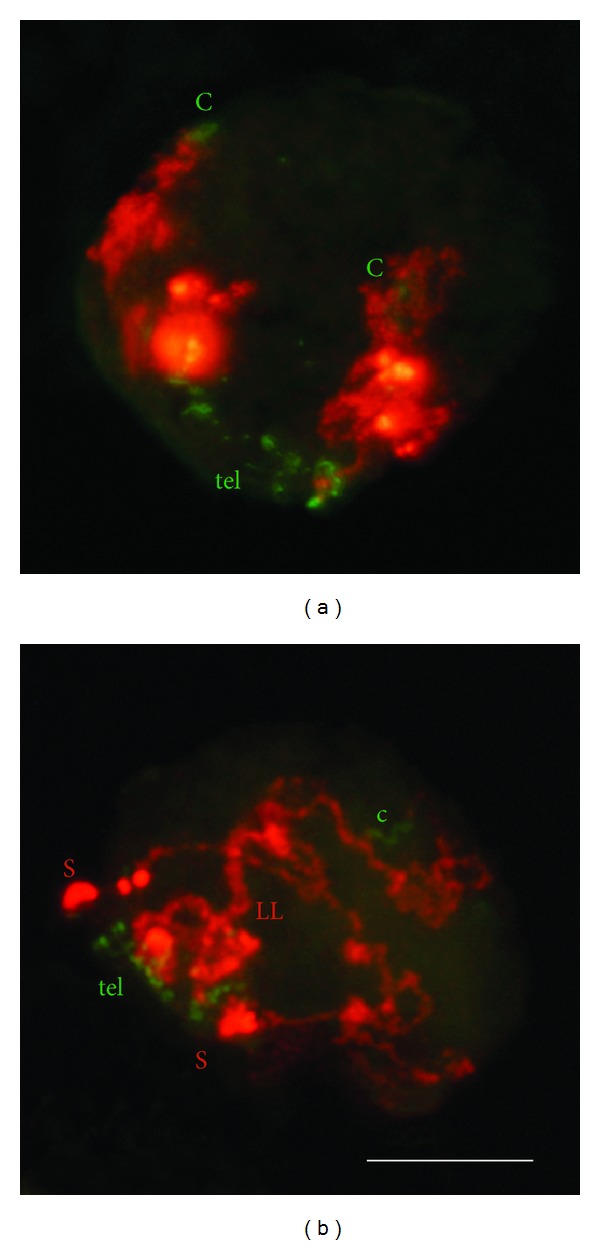
Arrangement of rye homologous chromosome pair 5R added to wheat at early meiosis. (a) Nucleus at leptotene showing highly compacted rye chromosomes located in separate territories. Chromosomes show the Rabl orientation with centromeres (c) and telomeres (tel) in opposite poles. Telomeres of wheat chromosomes, also labeled, are arranged in groups denoting the initiation of bouquet organization. (b) Nucleus at early zygotene showing partial synapsis of the long arm (LL) while the short arm ends (S) remain separate. The chromosome length is increased relative to leptotene, which causes that chromosomes span the entire nucleus. The centromere of one chromosome is out of focus. Bar represents 10 *μ*m.

**Figure 4:**
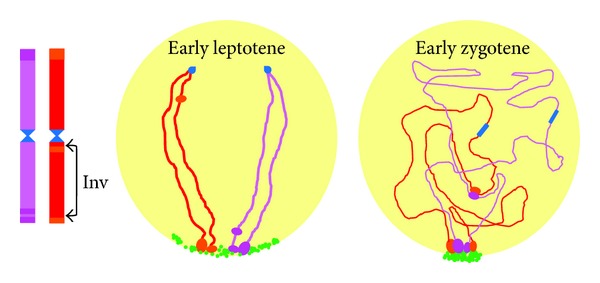
Diagrammatic representation of two homologous chromosomes with an inversion (inv) in heterozygous condition, in early meiosis. The positional change of a heterochromatic chromomere from distal to proximal denotes the length of the inversion. The normal and inverted homologous arms show an antiparallel orientation in early leptotene. In early zygotene, the subdistal chromomere of the normal arm pairs with its proximal counterpart in the inverted chromosome after movements concurrent with chromosome elongation.

The level of homologous pairing and synapsis produced in bouquet defective mutants of budding yeast or maize [25, 46] as well as in meiocytes where bouquet formation is inhibited by colchicine [38] indicates that telomere clustering is not absolutely necessary for pairing. Although telomere clustering may reduce the distance between homologues and facilitate their recognition, this convergent force affects mainly distal chromosome regions, especially in large chromosomes with lengths at zygotene of the order of tens or even one hundred *μ*m. Extensive chromosome pairing of large chromosomes of *Allium* species suggests that attraction forces between homologues operate on different points distributed evenly through most of the chromosome length [47, 48]. Chromosome movements that make possible encounters between intercalary regions are likely derived of chromatin conformational changes leading to chromosome elongation (Figure 3), which are produced concomitantly with telomere clustering at the leptotene-zygotene transition [15, 38]. Wheat and rye chromosomes multiply their length fivefold in leptotene relative to premeiotic interphase. Because the size of the nucleus remains the same at the leptotene-zygotene transition, or is even reduced, chromatin unfolding obliges chromosomes to move and span the entire nucleus. This chromatin unfolding is not inhibited by colchicine [38] and is therefore independent of telomere migration. The movement that chromosome elongation generates can facilitate the occurrence of chance collisions between homologous chromosomes. The reduction of long distances between homologues during zygotene is particularly apparent in the case of heterozygotes for an inversion covering almost 95% of a rye chromosome arm. Observations were made in a line of wheat with the disomic addition of chromosome 1R [49] and, therefore, in nuclei with more than 20 *μ*m in diameter. During bouquet formation the subdistal part of the normal arm locates at the telomere pole while its homologous counterpart in the inverted arm is situated at the centromere pole. However, such regions pair and synapse in many meiocytes. Collisions between distant homologous regions can be produced after chromosome elongation as indicated in Figure 4.

Life imaging of nuclear dynamics in maize meiocytes has shown that chromosome movements at zygotene include rotations of the entire chromatin and movements of individual chromosome segments [50]. Whether these movements were destined to promote pairing or were produced after synapsis was not established. However, movements of individual chromosome segments are expected to occur during chromatin unfolding. In addition recombination *per se* can increase the chromosome mobility. Rapid chromosome movements produced at early prophase in budding yeast are modulated by recombination. Mutants with defects in recombination do not show the most robust and fastest movements produced in the wild type [33]. On the other hand, in vegetative cells of budding yeast, either a given induced DSB or several DSBs induced by *γ*-irradiation trigger the mobility of all chromosomes as a component of the repairing mechanism [51, 52]. DSBs are potent triggers of the DNA-damage response, a complex signaling network involved in the activation of cell-cycle checkpoint kinases that halt the cycle until the damage is repaired. The chief transducer of the DSB signal is the nuclear protein kinase ataxia-telangiectasia mutated (ATM), which phosphorylates different substrates [53] that may lead to changes on chromatin organization and chromosome dynamics. The conformational change of chromatin leading to chromosome elongation at the end of leptotene is not a result of the DNA-damage response activated by DSBs produced by Spo11; during prophase I, the length of chromosomes is similar in both wild type and null *spo11* mutants in *Arabidopsis* and mouse [54, 55].

## 4. Centromeric Connections

Functionally equivalent domains, other than telomeres, present also in all chromosomes are the centromeres. They are the regions involved in attaching chromosomes to microtubules and that mediate chromosome movement during the cell division. The enrichment of different repeated DNA sequences, or proteins involved in their organization, may represent a possible source of chromosome interactions in finding the homologous partner. This possible role has been emphasized by the presynaptic centromere pairing observed in a wide range or organism including fungi, plants, and animals (reviewed by [56]), particularly in allopolyploid wheats [57, 58]. There are two ploidy levels, tetraploid and hexaploid, among polyploid wheats. Tetraploid wheat, *Triticum turgidum* (macaroni wheat), carries four sets of seven chromosomes (2*n* = 28, genome formula AABB). Genomes A and B derived from two related wild diploid progenitors, *T. urartu* and *Aegilops speltoides*, respectively. Bread wheat, *T*. *aestivum*, is a hexaploid species (2*n* = 42, AABBDD) that carries the A and B genomes of tetraploid wheat and the D genome of the wild diploid progenitor *T. tauschii*. In spite of the genetic affinity between chromosomes of genomes A, B, and D (homoeologous chromosomes) bread wheat forms 21 bivalents at diakinesis and metaphase I. The exclusive formation of homologous bivalents is the result of the suppression of homoeologous pairing principally controlled by the *Ph1* (pairing homoeologous) locus on the long arm of chromosome 5B [59, 60]. A region of 2.5 Mb containing a heterochromatin segment inserted into a cluster of cyclin-dependent kinase Cdk2-related genes was reported to be the *Ph1* locus [61]. However, its mode of action remains to be elucidated.

Germ cells of wheat enter meiosis with centromeres associated on average in pairs. This arrangement suggested that centromeres were involved in the mechanism of chromosome sorting and that homologous bivalent formation started with the premeiotic association of their centromeres promoted by *Ph1* [57]. The identification of centromeres of two homologous chromosomes of rye added to wheat demonstrated that centromere associations were mainly nonhomologous and independent of *Ph1* [15]. Centromeres cluster in more complex structures during leptotene of tetraploid and hexaploid wheats. Seven presynaptic multicentromere complexes were found in some meiocytes. This suggested the hypothesis that both homologous and homeologous centromeres, paired or unpaired at premeiotic interphase, become associated prior to synapsis and that, after homology recognition under the control of *Ph1*, each multicentromere structure is resolved in pairs of homologous centromeres [58]. This hypothesis implies a strong increase of homologous centromere association prior to synapsis and the presence of sufficient chromosome-specific DNA sequences to discriminate between homologues and homoeologues. This seems unlikely because centromeres usually contain abundant repeats that are not unique to individual chromosomes.

Homologous rye centromeres present in different wheat-rye disomic additions, in wheat-rye Robertsonian translocations, or introgressed into wheat chromosomes, are confined to spatially separated centromere clusters in more than 80% of meiocytes at early leptotene [56]. This happens regardless *Ph1* was present or not. The frequency of association of homologous centromeres increases with the progression of prophase I and this is a result of synapsis expansion. Thus, presynaptic centromere clustering in wheat is not based on homology and, therefore, cannot promote recognition of homologous chromosomes. This is consistent with the fact that pairing and recombination of homologous arms are not affected by heterozygosity for the centromere, as well as with the fact that homologous centromeres do not cause pairing of homoeologous chromosomes [62]. A FISH analysis of the behavior of centromeres and distal chromomeres in telocentric and biarmed chromosomes confirmed that it is not the centromeric, but rather the subtelomeric, regions that are involved in the search and recognition of the homologous partner [62].

In budding yeast wild-type meiosis, chromosomes become associated in pairs at their centromeres independent of chromosomal homology. Most centromere couples are nonhomologous at a stage before synapsis but undergo switching until all of them involve homologues [63]. This transition from random to homologous pairing requires Spo11 activity indicating that the partner change is dependent on the same recombination-based mechanism that regulates homologous pairing and synapsis. Early nonhomologous centromere pairing depends on the synaptonemal complex protein Zip1, which bridges the space between the cores of homologous chromosomes, but it is independent of Zip2 and Zip3, which are component of the synaptonemal complex lateral element. Homologous alignment is not affected in a *zip1* mutant although the synaptonemal complex is unable to zipper up [64]. This indicates that initial centromere interactions are dispensable for homologous chromosome pairing. It is likely that, in yeast too, synapsis expansion through chromosome arms brings together the homologous centromeres that were initially involved in nonhomologous pairs. This assumption is in agreement with the initiation of synapsis at the sites of crossover recombination [65]. However, homologous synapsis initiates also at centromeres after depolymerization of Zip1 bridging nonhomologous associations [66]. The molecular basis of the connection between nonhomologous centromeres remains to be established in wheat. However, the formation of multiple associations while yeast displays a pair-wise pattern can be explained by differences in the size of the centromere region. The large size of the wheat centromere can facilitate multiple interactions. Presynaptic centromere association does not appear as a relevant component of the chromosome pairing mechanism. Accordingly, in *Drosophila*, where homologues are paired at the onset of meiosis, centromeres cluster in oocytes at early zygotene. This centromere clustering appears to define the first step in the initiation of synapsis [67].

In organisms where homologous centromere pairing is driven by synapsis, other potential roles have been suggested for presynaptic centromere clustering. Centromere clustering is concurrent with telomere migration and chromosome movements derived from chromosome elongation produced during leptotene and early zygotene. Anchoring the chromosomes by this mechanism may provide stability to the centromere pole, reduce the disorder degree introduced by chromosome dynamics, and orientate properly the telomeres convergence [62]. In *Drosophila*, humans, and budding yeast nondisjunction events at the second meiotic division are enriched in centromere-proximal crossovers [68–70]. These proximal crossovers probably disrupt the cohesion of the pericentromeric region causing premature separation of sister chromatids at anaphase I and random segregation at anapahase II. Early centromere clustering might reinforce the effect of factors, such as heterochromatin, that suppresses proximal crossovers [71]. Finally, early nonhomologous centromere association has been suggested to be related with the formation of meiosis-specific kinetochores, which are arranged side by side and coorientate to the same pole at anaphase I [72].

## 5. Mechanisms for Homologous Recognition

### 5.1. Recombinational Interactions between Homologues

The mechanism by which homologous chromosomes recognize each other and pair varies between organisms. In many eukaryotes, homologous recognition depends on DNA/DNA interchromosomal interactions produced as a result of the initial repairing steps of Spo11-induced DSBs. Following the resection of the 5′ DNA ends at the breaks, the resulting 3 single-stranded DNA invade the intact DNA duplex of a homologous chromosome, as catalyzed by Rad51 and Dmc1 recombinases, and generate intermediates capable of assessing homology. In some fungi, the initiation of recombinational interactions is required for homologous alignment. In budding yeast, the assessment of homologous pairing by means of both Cre/*LoxP* and FISH assays in wild type, in the *spo11* and* ndj1* single mutants, and in the *spo11 ndj1* double mutant, yielded pairing values somewhat lower in the *ndj1* mutant, and much more lower in both the *spo11 *single mutant and the *spo11 ndj1* double mutant, than in the wild type. The absence of Spo11, regardless the bouquet is formed or not, causes the same effect on pairing [73]. This result indicates that meiotic telomere reorganization is not a DSB-independent component of the pairing mechanism and exerts its effect on homologous juxtaposition through its role in meiotic recombination. Electron microscopy three-dimensional analysis was used in *Sordaria* to follow the spatial relationships between chromosomes for all pairing stages in wild type and mutants [13]. Homologous chromosomes are aligned at the end of leptotene at a distance of 400 nm, prior to the bouquet formation. In the absence of Spo11, homologous axial elements show only rare signs of recognition and do not form synaptonemal complexes. However, after DSBs were induced by irradiation in the null *spo11* mutant, homologues become aligned. The number of paired segments is related to the number of DSBs measured by Rad51 foci that appear at the pairing sites. These results indicate that the recombination-dependent homologous recognition is an important component of the homologous pairing mechanism.

Rapid chromosome movements produced in meiotic prophase I, in budding yeast, require the attachment of telomeres to the nuclear envelope and frequently exceed 1 *μ*m/s. Although these movements, initially, are independent of recombination, they are qualitatively different in mutants showing defects in recombination, which suggest that chromosome kinetics responds to molecular changes in the recombination machinery [33]. Mutants in genes that link telomeres to the cytoskeleton through the intact nuclear envelope show a gradual variation in the chromosomal movement intensity. Pairing rates are directly correlated with the force of the motor activity, suggesting that rapid chromosome movements promote homologous collisions and pairing [74]. This is consistent with the fact that short chromosomes frequently remain unsynapsed when long chromosomes have finished synapsis; interactions are expected to occur more often between longer chromosomes.

The dynamics underlying the homologous search has been explored *in vivo* using two tagged homologous loci in vegetative cells of budding yeast [51]. These two loci occupy largely separate nuclear regions and move independently in a confinement space representing 2.7% of the nuclear volume in the absence of DNA damage. After the induction of a DSB these two loci pair ten times more often. There is a strong increase in the mobility of the locus in the cut chromosome, which can explore a nuclear volume ten times larger. The mobility of the signal in the uncut chromosome increases too; this locus explores a nuclear volume four times larger. The increase in the chromosomes mobility is dependent on the Rad51 recombinase and on Sae2, a protein involved very early in the processing of DNA ends. These results support a model for homologous pairing based on the activation of the homology search machinery by the detection of a DSB. During the homology search, the broken molecule moves into the nucleus checking for homology among sequences that it encounters until it finds a partner. Activation of the homology search mechanism greatly increases the nuclear region to be explored through DNA/DNA interactions. A similar mechanism might be activated by Spo11-induced DSBs in meiotic cells. Several DSBs repaired along each homologous pair would lead to their longitudinal apposition. The molecular events of recombination are thought to take place in cytological structures called recombination nodules. The number and distribution of early recombination nodules agree with this mode of homology recognition [75].

**Figure 5:**
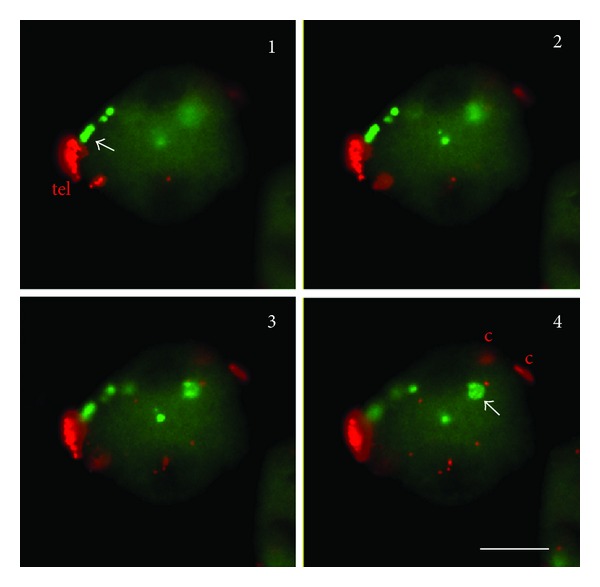
Topological arrangement of the telomere cluster (tel), rye centromeres (c), and the subtelomeric heterochromatin chromomere (arrows) of the short arm of chromosome pair 5R added to wheat in a 3D section stack. Only one of the 5RS telomeres entered the bouquet the other is in the middle of the nucleus close to the heterochromatic chromomere. Bar represents 10 *μ*m.

 In budding yeast, movements generated or increased by meiotic DSBs are most likely of sufficient magnitude to produce collisions, strand exchanges, and pairing between homologous sites initially located at any position in the nucleus. In eukaryotes with large genomes and nuclear volumes ten or more times larger, such movements are expected to be insufficient to complete pairing between all homologous pairs, hence homologues become aligned and initiate synapsis after bouquet formation [56]. A feature of the meiotic bouquet evolutionarily conserved is that its organization is independent of recombination since telomeres cluster in mutants of mice, budding yeast, or *Sordaria* lacking Spo11 [76–78]. In budding yeast and *Sordaria*, anchoring of telomeres to the nuclear envelope is required to generate a chromosome dynamics capable of achieving homologous identification before bouquet is consolidated. In many higher eukaryotes bouquet formation reduces long distance spacing between homologues making their identification possible. This has been demonstrated in the case of the rye chromosome pair 5R present in a wheat-5R addition line. While the wheat genetic background produces normal chiasma frequency, the behavior of chromosome 5R is conditioned by its submetacentric conformation. Migration of the telomere of the 5RS arm during bouquet formation is often delayed or incomplete [45]. This anomalous telomere dynamics (Figure 5) is followed by failure in pairing, synapsis and chiasma formation in the corresponding arm pair, which demonstrates that telomere clustering enhances the efficiency of the homologous search machinery to ensure meiotic pairing.

In many plant species, synapsis starts at sites close to the ends and succeeded by additional intercalary initiations to complete the synaptonemal complex assembly. Chiasmata are also nonrandomly distributed and locate in the distal half of chromosomes. In wheat, studies using deletion lines suggested that chiasmata occur mainly in regions with a high density of genes [79, 80]. That it is not the position but the DNA sequence, or chromatin organization, normally present in the distal part of a given chromosome arm that determines the crossover formation has been demonstrated in wheat and rye using inversions that change the position of crossover-rich regions from distal to proximal. The pattern of chiasmata is inverted in the inversion homozygotes [81, 82]. In inversion heterozygotes, the dynamics of the normal and inverted arms during zygotene demonstrates that crossover-rich regions are more active in recognizing the homologous partner and developing synapsis than crossover-poor regions [49]. In normal homozygotes, the crossover-rich regions are positioned in the vicinity of chromosome ends and their association is facilitated by telomere clustering. In inversion heterozygotes, crossover-rich regions are positioned centrally in one chromosome and distally in the homologue, and the same happens with the crossover-poor regions. However, crossover-rich regions pair more often. It is likely that the dynamics generated by chromosome elongation facilitates collisions between homologous sites of two antiparallel chromosome arms but the pairing results suggest a higher mobility of the crossover-rich regions. The question arises whether the intrachromosomal differentiation in the ability to find a match is related or not to the pattern of DSBs. However, there is no data on the distribution of DSBs in wheat and rye chromosomes. A high-resolution map of meiotic DSBs across the genome has been constructed only in two eukaryotes, *S. cerevisiae* [83] and mice [84]. In both species, the DSB map displays reasonable agreement with the crossover distribution map.

### 5.2. Nonrecombinational Interactions between Homologues

*Drosophila *and *C. elegans *must have recombination-independent mechanisms that lead to chromosome sorting and pairing since normal homologous synapsis occurs in mutants that have blocked DSBs formation [85, 86]. Chromosome rearrangements in *C. elegans* have shown that each chromosome contains a cis-acting sequence located near one end that aid homologous identification and is necessary for homologous recombination and segregation [87]. These sites are called pairing centers and interact with a family of four paralogous proteins, each containing two atypical C2H2 zinc fingers. Each protein localizes to the pairing centers of one or two chromosomes pairs during early meiotic prophase: ZIM-1 on chromosomes II and III, ZIM-2 on chromosome V, ZIM-3 on chromosomes I and IV, and HIM-8 on the X chromosome. Short sequence motifs enriched in the corresponding cis-acting element selectively recruit these proteins *in vivo*. Insertion of a cluster of recruiting motifs into a chromosome lacking its endogenous pairing centre is sufficient to restore homologous pairing, synapsis, crossover recombination, and segregation. Homology has been proposed to be checked at unique sequences interspersed with and/or adjacent to the major clusters of binding sites on each chromosome. Although *C. elegans* does not form bouquet, chromosome movements that bring homologous pairing centers together are generated by a mechanism similar to that of telomere clustering. These movements are mediated by the SUN-1/ZYG-12 (SUN-KASH) protein complex which spans the nuclear envelope and connect each pairing center with the microtubule motor protein dynein [88]. The complex formed by the pairing center and the transmembrane proteins SUN-1 and ZYG-12 moves chromosomes along the nuclear envelope using dynein-dependent microtubule forces.

Another pairing site has been identified in the sex chromosomes, in *Drosophila *[89]. This pairing center contains 200–250 tandem repeats of rRNA genes (also called rDNA) and is located in the heterochromatin of the X chromosome and near the base of the short arm of the Y chromosome. Deletion of the X chromosome rDNA causes failure of X-Y pairing and sex chromosomes nondisjunction. These meiotic abnormalities are partially restored in transgenic males with the insertion of a single copy of the rDNA in the deleted X chromosome. The pairing activity resides in a 240 bp sequence that is repeated in tandem arrays of six to ten copies in the intergenic spacer found between ribosomal genes. These 240-bp repeats function to recruit two proteins, SNM and MNM, which are required for stable homolog pairing and segregation in the male. Interactions between proteins of the sex chromosome appear to substitute for chiasmata in the achiasmate male meiosis. However, the binding sites of SNM and MNM proteins on autosomes remain undefined. Autosomes in both sexes and the two XX in the female are already paired at the entrance of meiosis. How this arrangement is achieved has been investigated by means of a high-throughput FISH technology that enabled a genome-wide RNAi screen for factors involved in somatic pairing. Both pairing promoting genes and anti-pairing genes have been identified supporting the idea that homologous pairing is mediated by a balance of factors with opposing functions [90]. The pairing promoters group comprises many genes with functions associated with mitotic cell division while genes involved in the S-phase progression, DNA replication, and chromatin compaction are included in the antipairing group. The molecular basis of the homologous recognition mechanism and how pairing is promoted or impeded represent an exciting challenge for the future research.

 A different homology-detecting mechanism in which RNA mediates homologous chromosome association during meiosis has been described in a recent report in fission yeast [91]. The arrangement of the fluorescently tagged *sme2* locus was monitored in live meiotic cells. The *sme2* gene encodes noncoding polyadenylated RNAs meiRNA-S and meiRNA-L, which enable cells to switch from mitosis to meiosis by binding proteins Mei2 and Mmi1. Mei2 is a positive regulator of meiotic entry that forms a distinct dot at the *sme2* locus on chromosome II in the prophase horsetail nucleus. Mmi1 binds to meiotically essential mRNAs and destabilizes them in mitotic cells. In zygotic cells, Mmi1 is recruited to the *sme2* site and inactivated by Mei2 permitting the progression of meiosis. The two Mei2 dots initially present in zygotic cells join each other immediately after karyogamy and remain paired during most of the horsetail stage. Robust pairing at the *sme2* locus occurs in the absence of recombination and requires transcription of the 1,5 kb meiRNA-L. The pairing activity locates at the 3′ of the meiRNA-L transcript and to be effective requires RNA transcripts from both chromosomes. The RNA-mediated pairing at the *sme2* locus suggests that RNA-containing complexes spread along the chromosomes may act as chromosome-specific identifiers. Although there is no evidence supporting a role of RNAs on meiotic pairing in other organisms, the notion that transcripts anchored in one chromosome can be used in the identification of homologous sequences seems feasible [92]. Contacts between homologous chromosomes centered on DSBs produced in active genes have been demonstrated in human somatic cells at G0/G1-phase [93]. These contacts are abrogated by the transcriptional inhibitors actinomycin D and *α*-amanitin, which indicate that transcription of coding-RNA can mediate homologous recognition.

 Other chromosomal features different from DNA/DNA recombinational interactions or RNA-mediated pairing have been proposed to be involved in the homologous recognition. This is the case of the pattern of cohesin distribution in the axial elements of unmatched meiotic chromosomes in mice [94]. Two meiosis-specific subunits, Rec8 and Rad21L, of cohesin locate at the axial elements in early meiotic cells. Complexes containing Rec8 or Rad21L are spatially separated and their pattern of distribution along the chromosomes axis is the same in the two homologues. These results led to the proposal that cohesin composition of axial elements could provide a code for homologous recognition.

## 6. Pairing Correction

The exploratory chromosome movements across the surrounding space to find a partner generate interactions between homologues but also between nonhomologues as supported by nonhomologous synapsis produced in a number of meiotic mutants in different organisms. This implies that the homology-identification mechanism should include some component with the function of destabilizing and dissociating nonhomologous collisions to reinitiate additional search attempts that culminate in homologous pairing. Little is known about the mode by which chromosomes detect and eliminate inappropriate interactions. A recent report has shown the timely concurrent disappearance of improper chromosome associations with the telomere cluster disorganization [49]. In heterozygotes for the almost complete inversion of a rye chromosome arm, improper and unstable associations involving terminal or proximal regions of the normal and inverted chromosomes disappear concomitant with telomere clustering dissolution. Meanwhile distal and proximal homologous regions continue to interact and undergo stable synapsis. This suggests that chromosome movements that disperse the chromosome ends during bouquet dissolution are involved in the correction of pairing. Such movements may keep apart nonhomologous chromosomes that initiate a new search to find the correct partner. The difference between stable and unstable associations, and, therefore, consolidation or correction of pairing, seems to depend on the capability that chromosomal contacts have to form a crossover or not.

Dissolution of telomere clustering concerns most likely the correction of pairing and synapsis produced in autotriploids and autotetraploids of the silkworm *Bombyx mori*, as well as in allopolyploid wheats. The females of *B. mori* undergo achiasmate meiosis while males form chiasmata and display the standard meiotic pathway. In the triploid females, homologous chromosomes form trivalents in zygotene which are converted in bivalents plus univalents by the end of pachytene. A similar synaptic correction occurs in the tetraploid females; each quadrivalent formed at zygotene is transformed in two bivalents at pachytene. However, in autotriploid and autotetraploid males, multivalents are preserved until metaphase I [95–97]. In allopolyploid wheats, chromosomal interactions produced at early zygotene lead to homology detection between homologues and also between homoeologues, which gives rise to a synaptic pattern characterized by the presence of multivalent synaptonemal complex configurations formed by both homologues and homoeologues, in addition to homologous bivalents [98–100]. Such multivalents are reduced to homologous bivalents at late zygote and pachytene. This pairing correction mechanism is under the control of the homoeologous pairing suppressor *Ph1. *In the presence of *Ph1*, chiasmata are formed only between homologous chromosomes but, when *Ph1* is absent, chiasmata can also be formed between homoeologues and multivalents persist until metaphase I [101, 102]. Thus, chiasmata condition also the dynamics of pairing and synapsis in polyploid wheats and silkworm.

 The molecular basis of the bouquet disorganization is poorly understood. In budding yeast meiosis, the absence of the Rec8 cohesin subunit inhibits exit from telomere clustering and disrupts spindle-pole body and telomere cluster colocalization [103]. Expression of the mitotic cohesin component SCC1 from a *rec8* promoter in meiotic cells restore these defects, indicating that cohesin mediates the association of the telomere cluster and the spindle-pole body and exit from the bouquet stage. On the other hand, telomere-led rapid chromosome movements, which are considered to foster homologous pairing [74], have been proposed also as a stringency factor for eliminating unwanted connections in budding yeast [104]. Accordingly, in *C*.* elegans*, chromosome movements mediated by the SUN-1/ZYG-12 complex, which bring together homologous pairing sites, also cooperate to inhibit initiation of synapsis between transiently associated nonhomologous centers.

 In diploid organisms errors in the homology search lead to nonhomologous pairing, but polyploid species such as tetraploid and hexaploid wheats have, in addition, the necessity of discriminating between homologous and homoeologous chromosomes in order to ensure a regular diploid-like meiotic behavior. Polyploid wheats evolved an efficient pairing correction mechanism, which is absent in related diploids and operates under the control of *Ph1. *A recent report reveals alterations in the course of premeiotic replication and an increased Cdk2-type phosphorylation of histone H1 in the absence of *Ph1* [105]. However, the connection of these effects with the occurrence of homoeologous pairing and recombination remains to be elucidated.
